# Principles of Medicine

**Published:** 1857-09

**Authors:** 


*Principles of Medicine. An elementary view of the Causes, Nature, Treatment, Diagnosis and Prognosis of Disease; with brief remarks on Hygienics or the Preservation of Health. By Charles J. B. Williams, M. D., F. R. S. A new American from the third and revised London Edition. Blanchard and Lea, Philadelphia*, 1857.

Former editions of this work have been several years in the hands of the profession, and are too generally known and appreciated to require an extended notice of the present issue. The volume before us contains about 500 octavo pages. In our estimation it is decidedly the best work on general Pathology to which the student can have access at the present time.

The analytical method of investigation pursued by the author in the preparation of this work, adds greatly to its value. It is the only true and rational method of studying disease, and we only regret that several important subjects are altogether omitted from this as well as from former editions of the work.

